# Tactics and Strategies for Managing Ebola Outbreaks and the Salience of Immunization

**DOI:** 10.1155/2015/736507

**Published:** 2015-02-10

**Authors:** Wayne M. Getz, Jean-Paul Gonzalez, Richard Salter, James Bangura, Colin Carlson, Moinya Coomber, Eric Dougherty, David Kargbo, Nathan D. Wolfe, Nadia Wauquier

**Affiliations:** ^1^Department of Environmental Science, Policy, and Management, University of California, Berkeley, CA 94720, USA; ^2^School of Mathematical Sciences, University of KwaZulu-Natal, Private Bag X54001, Durban 4000, South Africa; ^3^Metabiota, Inc., 1 Sutter Street, Suite 600, San Francisco, CA 94104, USA; ^4^Computer Science Department, Oberlin College, Oberlin, OH 44074, USA; ^5^Metabiota, Inc., 24 Main Motor Road, Congo Cross, Freetown, Sierra Leone; ^6^Metabiota Inc., Kenema Government Hospital, Kenema, Sierra Leone; ^7^Directorate of Disease Prevention and Control, DPC Ministry of Health and Sanitation, Freetown, Sierra Leone; ^8^Sorbonne Université, UPMC, Université de Paris 06, CR7, CIMI-Paris, 75005 Paris, France

## Abstract

We present a stochastic transmission chain simulation model for Ebola viral disease (EVD) in West Africa, with the salutary result that the virus may be more controllable than previously suspected. The ongoing tactics to detect cases as rapidly as possible and isolate individuals as safely as practicable is essential to saving lives in the current outbreaks in Guinea, Liberia, and Sierra Leone. Equally important are educational campaigns that reduce contact rates between susceptible and infectious individuals in the community once an outbreak occurs. However, due to the relatively low *R*_0_ of Ebola (around 1.5 to 2.5 next generation cases are produced per current generation case in naïve populations), rapid isolation of infectious individuals proves to be highly efficacious in containing outbreaks in new areas, while vaccination programs, even with low efficacy vaccines, can be decisive in curbing future outbreaks in areas where the Ebola virus is maintained in reservoir populations.

## 1. Introduction

Beginning with a hypothesized natural reservoir-to-human spillover of the Zaire ebolavirus (EBOV) in Guinea in February 2014 [1, 2], by mid-November this outbreak had grown to more 15,000 cases, which is at least thirty times larger than the largest Ebola virus disease (EVD) outbreak in recorded history [3]. Though small by pandemic standards, mortality rates around 30–90% [4, 5] make EBOV and allied filovirus of the same family some of the most feared pathogens in the world. Further, beside the social human cost, failure to control epidemics has catastrophic consequences for the economies of countries where major outbreaks occur [6] and substantial negative impacts on global travel and trade as well [7].

Current efforts to control the West African outbreak include, among other international agencies, deployment of WHO personnel and US Army units to help detect, isolate, and treat infectious individuals. The outbreak itself is much more complex than suggested by the models we build to predict incidence rates over time and appears to be governed by different frequency parameters in different regions of West Africa. For example, a differential equation model, fitted to incidence data for the current EVD outbreaks in West Africa, estimated *R*_0_ (the number of cases produced by each case at the start of the epidemic) to be 1.52, 2.42, and 1.65, respectively, in Guinea, Sierra Leone, and Liberia [8]. A related approach obtained an aggregated estimate of *R*_0_ = 1.78 across all of West Africa [9, 10]. These estimates, while useful, can be quite variable [11, 12]. Further, they do not inform us, for example, about the relative importance of early case detection versus effective isolation in managing the current outbreak. In addition, they also neglect to include structures that can easily be incorporated to provide assessments of the effectiveness of vaccination programs, presumably because no vaccines have yet been approved by recognized authorities.

In the modeling study presented here, our focus is threefold: (1) to demonstrate the range of variability that can be expected in terms of fadeouts (epidemic fails to materialize from a few initial cases: see [13, 14] for more details) and outbreak sizes, as EVD may spread from one region to another; (2) to provide an indication of the sensitivity of outbreak sizes and length of epidemics to changes in contact frequencies among susceptible and infected individuals, case detection rates, and isolation rates during the course of the epidemic; and (3) to evaluate the importance of developing a vaccine [15] for future control of EVD in terms of vaccine efficacy levels needed for a vaccination program to be effective.

## 2. Model

**Figure 1:**
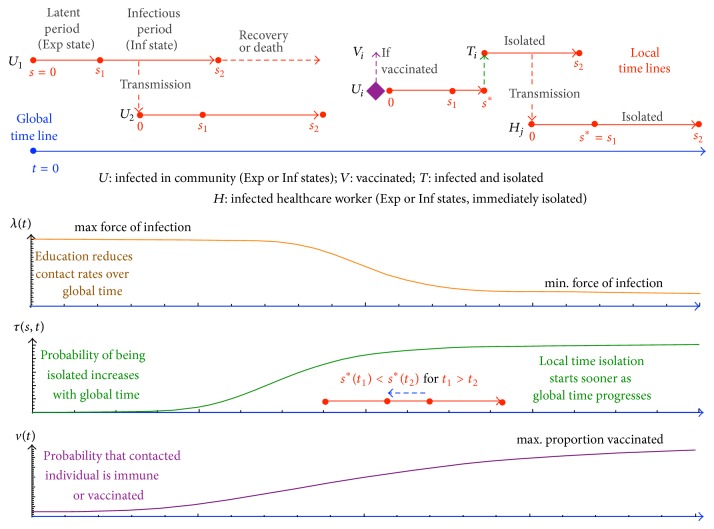
Our model is a Markov chain branching process in which an individual in state *U*_Exp_ (Exp: exposed/infected but not yet infectious state) can be generated from an individual in state* U*_Inf_ (Inf: infectious state) with probability 0 < *λ*_min⁡_ < *λ*(*t*) < *λ*_max⁡_ < 1, which is assumed to decrease with time as individuals in the community become more cautious about making casual contact with individuals that have Ebola virus-like symptoms (see SOI Methods for functional forms). Setting the local time of infection of this individual to *s* = 0, this individual becomes infectious at *s* = *s*_1_, which we assume to be constant, but can be treated as a random variable with a finite range distribution centered on *s*_1_ (e.g., a beta distribution). While infectious on the interval [*s*_1_, *s*_2_], this individual may contact and infect other individuals, say one at time *s* ^*^—provided this individual is not immune (recovered) or has not been vaccinated with probability *v*(*t*) increasing over time (see SOI). We assume the infected individuals* U*_Inf_ either die or recover and are immune at *s*_2_ units of time after being infected (this can also be made a random variable if desired). Here we illustrate several (ignoring Exp or Inf subscript) infected individuals: *U*_1_ the index case, *U*_2_ the first of the secondary cases, and *U*_*i*_, an arbitrary general case. Over global time, *t*, we assume that it becomes increasingly likely—with probability 0 < *τ*(*s*, *t*) < 1 (see Figure S1 in Supplementary Material available online at http://dx.doi.org/10.1155/2015/736507)—that any individual *U*_*i*_ is isolated from the community while in its Inf state, and it is then able to transmit only to healthcare workers and does so to an arbitrary healthcare worker *H*_*j*_. The dependence of this probability on *s*, as well as *t*, allows us to consider case detection efficiencies. Additional model assumptions include the following: isolated patients can only transmit to healthcare workers at a rate given by *λ*_min⁡_, and infected healthcare workers are isolated immediately on infection.

Here we build a Markov transmission chain model [16] that allows us to investigate the three focal issues mentioned above. To achieve this, our model, as outlined in Figure 1, distinguishes between infected and infectious individuals, as well as between infections arising in the community, from isolated individuals or to and from healthcare workers. Further, as depicted in Figure 1, it incorporates functions characterizing population and public health responses to the epidemic, including community responses, healthcare case detection, patient isolation, and vaccine interventions. We relegate the mathematical details of our model to supplementary online information (SOI) and we refer to a set of baseline parameter values that are provided in Table S1 (SOI). A typical run of our model produces either a fadeout or an outbreak (as discussed in Figure 1) in which the number of cases grows each week during the initial stages of the epidemic.

Simulations of our model allow us to estimate both fadeout rates [13, 14], which are associated with emerging epidemics in new regions (i.e., naïve population), and the range of epidemic sizes that we can expect in future outbreaks. They also allow us to address our focal issues of inherent variability in the size of epidemics, assessment of the sensitivity of outbreak sizes and length of epidemics to selected processes, and an evaluation of possible vaccine efficacy [15] on future outbreaks.

**Figure 2:**
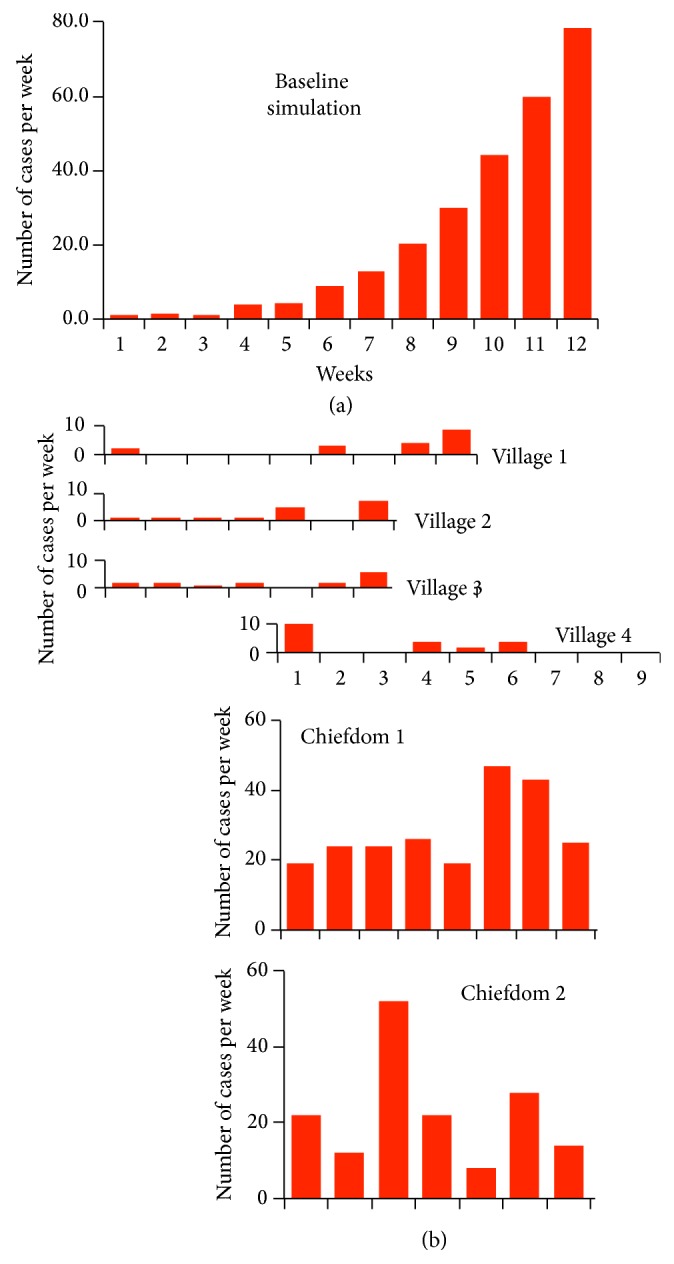
(a) A bar plot of the average weekly incidence rates during outbreaks (i.e., given that immediate fadeout did not occur) over 12 weeks, starting with an index case at the beginning of week 1, as generated from 100 runs of our transmission model, using the baseline parameter set in Table S1 (see SOI for details). (b) Plots of weekly incidence rates in 6 local areas (see Figure S2 for daily rates) that have been shifted to allow us to visually compare the shapes of these bar plots with model output.

We note that since estimates of *R*_0_ for outbreaks in different countries have ranged between 1.5 and 2.5 [8], it makes no sense to estimate model parameters from the data for one country and then apply the model to predict the course of an outbreak in another country. Additionally, the stochastic nature of outbreaks implies, as we demonstrate in our baseline simulation results below, that the same model parameters produce events that may either fadeout or breakout, where, in the latter case, the outbreak sizes may differ by nearly two orders of magnitude. Finally, even within country, as our data (Figure 2) and the data of others show (as discussed below), sufficient spatial structure exists so that a model not accounting for this spatial structure (e.g., rural areas versus towns and cities) cannot accurately forecast the course of any within country epidemic. Thus, the primary value of our model is to investigate, in the context of an ensemble of simulated events, the potential impacts of healthcare responses such as reductions in contact rates of community members with infected individuals or the efficacy of vaccination programs, should a suitable vaccine be released.

## 3. Incidence Data

In Figure 2, we illustrate the average weekly incidence over 80 such model-simulated outbreaks. We also present data on the incidence rates collected from 6 different locations in Sierra Leone between the period May 23, 2014, and July 14, 2014 (see SOI Methods and daily numbers plotted in Figure S3.).

The question arises whether any of these incidence data appear to be a self-contained local outbreak, in which a single case, transmitted from “outside-to-inside” can be regarded as the index case. In Figure 2, we see that Villages 1–3 could represent small local outbreaks, while Village 4 and Chiefdoms 1 and 2 have incidence patterns that would correspond to an earlier outbreak that could be fading (Village 4), an early outbreak that could be in its midstages (Chiefdom 1), or part of a bigger regional outbreak with infected individuals moving in and out of the village at a quite variable rate (Chiefdom 2). In reality, the epidemics in any one of these local areas is likely to be part of a greater epidemic that has considerable spatial structure. Inherent in this structure are heterogeneities in both transmission rates and susceptibility levels among individuals who are stochastically moving in and out of a collection of villages and small or larger towns that constitute a more self-contained region. Similar patterns have been found in Liberia [17], where, for example, the 983 cases in the Montserrado district roughly follow its own outbreak pattern from week 22 to 37 (the week starting Sept 8, 2014), while the 707 cases in Lofa during the same period indicate that Lofa must be a small part of a larger regional outbreak.

The spatial complexity indicated by our Sierra Leone data (Figure 2) and by comparable Liberian data [17] suggests that models assuming within-country spatial homogeneity are likely inadequate for making reliable predications on the course of the current outbreaks in Sierra Leone, Liberia, and Guinea. Future elaborations of our model to incorporate spatial structure require information on the rate at which individuals move among different regions [7, 18]. Given the lack of models with any spatial structure, our model currently provides the best available tool for obtaining insights into the importance of different tactics for managing EVD during the current outbreak, preventing off-shot EVD outbreaks in other countries, or from developing strategies for preventing future EVD outbreaks in the countries where Ebola virus exists in reservoir populations.

## 4. Baseline Simulation Results

Any outbreak is a single realization of an underlying stochastic process that exhibits considerable variability among repeated realizations of the same exact process. To demonstrate this variability, we repeated 20 simulations of the model, using a set of baseline parameter values (Table S1) obtained from a combination of estimates in the literature and tuning the results of our simulations to include the current epidemic as a possible realization of our model. From the results of these 20 simulations (Table 1), we see that the process failed to break out 20% (4 simulations) of the time: these are the “fadeout” rates that occur even when *R*_0_ > 1 [13, 14]. The number of total cases throughout the course of the simulated epidemic exceeded 1,000 in half of the remaining runs (8 simulations) but reached a cumulative total of less than 1,000 in the other half. The largest and smallest simulations yielded total cases differing by a factor of nearly 30 (3627/128 = 28.3). The direct calculations of the mean of the offspring distributions (distribution of the number of new cases produced by each case) of those individuals dying or recovering in the first 50 days provide an estimate of *R*_0_. We see in Table 1 that estimates of *R*_0_ are rather variable because of small population sizes and the demographic stochastic effects arising from the fact that transmission is a Bernoulli variable [19] (cf. Figure 1). For example, though we obtained much larger outbreaks with a slight modification of parameters, the largest of the twenty simulated outbreaks in Table 1 is 3,627 cases (Run 17), its initial offspring distribution was calculated from 26 cases, and it had a mean value of *R*_0_ = 2.48. By contrast, another realization of the same epidemic process (Run 19, Table 1) produced a total of 454 cases and its initial offspring distribution was calculated from only 9 cases and had a mean value of *R*_0_ = 1.76 (Table 1). Using Althaus's method [8] to estimate *R*_0_ from the incidence data produced by these two simulations, we obtained *R*_0_ = 3.03 (cf. Table 1 Run 17, *R*_0_ = 2.48) and *R*_0_ = 2.21 (cf. Table 1, Run 19, *R*_0_ = 1.76). Since both runs are realizations of the same stochastic process, these results support the reservations of some researchers concerning the utility of *R*_0_ as an index of epidemic intensity [20, 21], at least if it is estimated during the early stages of any outbreak.

Our results in Table 1 indicate that, as simulated outbreaks proceed, the value of *R*_0_ (which we calculate directly from the offspring distributions: it is just the means of these distributions) decreases over consecutive intervals of time and ultimately falls below 1.0 as the epidemic burns out (either due to a decreases in contact rates or a decrease in the probability of transmission-per-contact due to interventions or changes in the behavior of individuals). This is clearly depicted in Table 1, where, across the 20 runs, the values of *R*_0_ are relatively consistent across the larger epidemics in the sampling periods 51–100 days (*R*_0_ ≈ 1.5–1.7), 101–151 days (*R*_0_ ≈ 0.9), and 151–200 days (*R*_0_ ≈ 0.5). Note that implicit in our model is the assumption that the proportion of infected individuals in the total population remains negligibly small throughout the epidemic (i.e., less than 1%). Surprisingly, the value of *R*_0_ as whole for each simulated outbreak is 1.00 when rounded to two decimal places, except for the smallest outbreak in Table 1 where it is 0.99. This level of consistency from a stochastic process was not anticipated by the authors, particularly given the variability in the initial estimates of *R*_0_ from the twenty different realizations recorded in Table 1 of repeated realizations of the same Markov-chain pathogen-transmission process. Also, the distribution of lengths of epidemics is more consistent than the distribution of the total cases recorded in Table 1, ranging between only 177–240 days, despite a near 30-fold difference in the size of the outbreaks. This result holds because the length of the outbreak is strongly affected by the time course of the functions in our model that characterize changes in the background transmission rate (*λ*(*t*): see SOI) and in the healthcare response (*τ*(*t*, *s*): see SOI). Besides the number of cases, length of epidemic, offspring distributions, and associated *R*_0_ (the latter two over selected periods of time), each run of the model can also be used to compute new infections per day, incidence curves, new isolations per day, number in isolation facilities day by day, and so on (Figure S1), thereby providing estimates of resources needed under different intervention strategies.

## 5. Alternative Simulation Results

As a note of caution, the results presented in Table 1 pertain to a community that has a learning response parameter of 100 days (the time it takes for contact rates in the community to drop from a maximum level to halfway between the maximum and minimum levels, as the community adapts to reducing transmission during the course of the epidemic: cf. the *λ*(*t*) curve in Figure 1), which is our baseline value. This learning response parameter appears to have higher values in the current West African epidemic, so by way of illustration we also ran 100 simulations with this parameter set to 350 days. In these simulations the outbreaks grow more slowly but are much larger on average than those depicted in Table 1. In several of these runs, the outbreaks exceeded 20,000 cases within six months after initiation of the index case. This compares with estimates that by the start of November the number of cases in the current West African outbreak will exceed 20,000 [2]. Additionally, our simulations indicated a doubling time around the 120-day mark of 28 days. Assuming case estimates are accurate [2], this compares with a 26- to 27-day doubling time for the current West African epidemic over the month of September 2014 (SOI Table S3).

We also evaluated the effect of increasing the detection rate parameter by carrying out 100 simulations with the baseline parameter values, but changing the detection rate parameters in the function *τ*(*s*, *t*) from *c*_3_ = 3 to *c*_3_ = 1 and *c*_4_ = 250 to *c*_4_ = 500. The effects of these parameter changes on the probability of isolating cases are considerable, as illustrated in Figure S1 (SOI). For example, 100 simulations of the baseline parameter case corresponds to a 6% probability of isolating a case halfway through his or her infectious period on day 100 of the epidemic, while the 100 simulations of the alternate case corresponds to a 63% probability for the same time parameters. This increase in probability of early isolation curtailed all outbreaks to fewer than 1000 cases (cf. 50% of outbreaks exceed 1000 in Table 1). More importantly, however, the expected size of the 76 outbreaks that occurred (24 of the simulations were fadeouts with fewer than 10 cases) now fell to 235 cases (mean duration 165 days), with only 5 of 76 outbreaks exceeding 500 cases. Beyond repeat simulations of our model with various parameter values to obtain statistic on fadeout rates, size, and duration of epidemics, we use it to obtain estimates for the proportion of index cases that fadeout compared to those that breakout, as well as the expected number of cases and duration of the epidemic.

In addition, they allow us to construct offspring distributions for different phases of the epidemic across different realizations and transmission tree structures [22, 23] that might provide clues to the role of superspreaders [14], or other heterogeneities in the susceptibility and infectiousness of individuals (e.g., deceased patients and unsafe burial ceremony). Information on offspring distributions can also be used to help fit models to single outbreaks [24]. From Table 1 we see that estimates of *R*_0_ are very robust across epidemics differing in size by more than an order of magnitude, provided the offspring distributions are sufficiently large, which typically holds except for the initial and final stages of an epidemic.

## 6. Model Fitting Considerations

With the rapidly increasing power of genetic sequencing methodologies, transmission trees can be constructed for viral pathogens, such as EBOV [25], using genetic data [12, 22, 26, 27]. Thus, despite being a very challenging problem, the key to fitting a stochastic process model to a single realization, represented by a particular outbreak, appears to be rooted in fitting the model to the associated offspring and phylogenetic tree distributions that emerge and that better characterize the actual process [23, 24] than the much more variable case size or *R*_0_ statistic. It has recently been reported that EBOV genomes were sequenced, using blood samples from 78 patients in Sierra Leone [25]. Although these data were informative regarding the origin of the epidemic and in estimating viral mutations rates during the course of the current epidemic, they were insufficient for constructing transmission trees using newly developed Bayesian methods [28]. Obtaining reliable transmission trees from genetic data is a daunting task. However, obtaining sufficient data to estimate offspring distributions for windows of time during the course of an epidemic is within the realm of current technology, particularly if accurate contact tracing or, at least, spatiotemporal incidence records can be used in conjunction with pathogen genome data to infer likely offspring relationships.

Although we have insufficient offspring distribution data at this time to fit our model to the current EVD West African outbreak, our baseline parameters generate realizations that are compatible with past and current EVD outbreaks. To assess the sensitivity of our model to selected perturbations in our baseline data, for the purposes of illustration we generated a set of realizations with initial transmission risk rate reduced from *λ*_max⁡_ = 0.30 to 0.23. At the same time, also for purposes of illustration, we increased the learning response parameter from 100 (baseline value) to 200 days. In comparing the baseline parameters (referred to as Params1) and this alternative case (referred to as Params2; cf. Table 1 and Table S2), the initial outbreak is more likely to fadeout than breakout into a full-blown epidemic using Params1. When epidemics do breakout, however, they are larger in the case of Params2 (cf. 7958 versus 3627 for largest epidemics in each set of runs) and last for a longer time (average 310 days versus 224 days for epidemics >1000 cases). This sensitivity demonstrates the importance of looking at distributional structures, such as offspring distributions, during the course of an epidemic when it comes to assessing both the likely size and duration of an ongoing outbreak.

Given that we cannot currently decide, with the data we have, whether Params1 or Params2, or another set of similarly valued parameters, provides the most reliable fit of our model to outbreaks in Guinea, Liberia, Sierra Leone, or elsewhere, we can, at least, use our model to qualitatively evaluate the response of a particular outbreak to both tactical and strategic interventions. In particular, we use our model to assess the effectiveness of vaccination programs in preventing future outbreaks.

## 7. Vaccination Strategies

**Figure 3:**
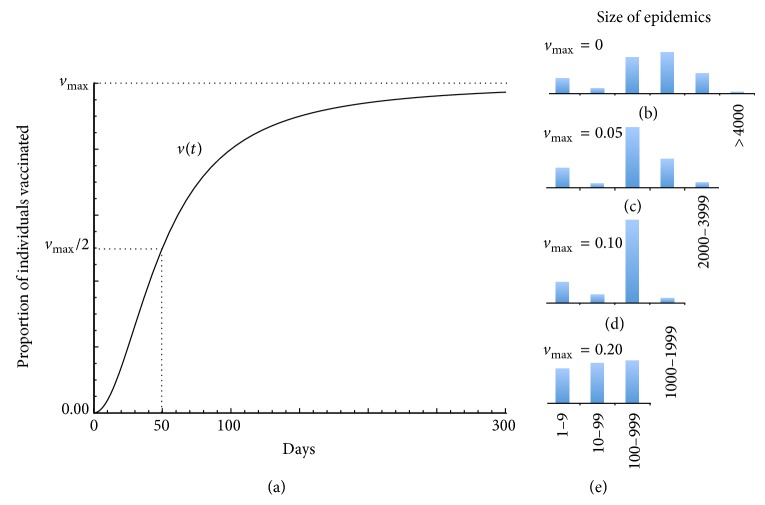
(a) Proportion *v*(*t*) of individuals vaccinated is plotted over 300 days. (b)–(e) Histograms (proportions in each size class sum to 1) of epidemic sizes (number of cases) over 100 repeated simulations, using the basic parameters (Table S1) with values for *v*_max⁡_ as specified. (b) When *v*_max⁡_ = 0 (no vaccination), outbreaks range from >4000 through a mode of 1000–1999, a mean of 1263 cases and a small number of fadeouts (category 1–9 cases). (c) When *v*_max⁡_ = 0.05, the mode is now in the 100–999 range and the mean is 759 cases. (d) When *v*_max⁡_ = 0.10, very few outbreaks exceed 999 and the mean is 350 cases. (e) When *v*_max⁡_ = 0.20, the cases are now almost equally distributed in the lowest three categories, and the mean is 117 cases.

Candidate vaccines exist, as well as therapeutic approaches, and are undergoing early trail evaluations: calls for their use in the current epidemic have been made [15]. Using Params1 (Table S1), we assessed the value of rolling out a vaccination program in which coverage is zero at the start of the epidemic and gears up slowly initially (first two weeks) but then more rapidly to reach half the maximum coverage rate by day 50, after which it rises more slowly again to asymptotically approach the maximum coverage rate as the epidemic progresses (Figure 3(a)). We compared four cases (Figures 3(b)–3(e)) in which the maximum coverage rates were 0% (control case, panel (b)), 5% (panel (c)), 10% (panel (d)), or 20% (panel (e)). These produced reductions of 40%, 72%, and 91%, respectively, relative to the no vaccination (control) case (for a similar analysis in the context of measles in sub-Saharan Africa see [29]). We performed a similar analysis using Params2 and found that a 10% maximum vaccination rate reduced the expected outbreak size by 83% (from 1569 to 261 cases: see Figure S4). We note that this analysis applies to both vaccination coverage of a 100% effective vaccine at the indicated levels and vaccination coverage at greater levels using a vaccine that is not 100% effective. Thus, for example, *v*_max⁡_ = 10% could pertain to 20% coverage using a vaccine that is only 50% effective.

## 8. Conclusion

Real epidemics are considerably more heterogeneous than is suggested by the model we developed here. First, incubation and infectious periods are not constant, but they have lengths that are better represented by random variables distributed over some finite range of values [30]. Second, heterogeneity occurs at the individual level with regard to likelihoods that some individuals transmit pathogens (e.g., individuals may be superspreaders for physiological or behavioral reasons) [14] or succumb to infection (due to both environmental and genetic factors) more than others [31]. Third, pathogen strain diversity can lead to considerable complications that have been comprehensively discussed in the context of many diseases [27, 32]. Fourth, both reservoir hosts and pathogens evolve over time, so that no two epidemics separated in space or time are likely to be driven by identical underlying transmission processes [33], and metapopulation structure itself plays a crucial role [34]. Fifth, individuals move around, and spatial processes can often critically influence outbreak patterns [7, 18]. Clearly, only individual-based models can be refined to account for all of these different kinds of heterogeneity. In particular, with regard to our second point, the existence of asymptomatic EVD cases has been shown to occur in previous outbreaks. If such cases are not explicitly accounted for, models will tend to overestimate the size of resulting outbreaks [35]. We can account for this phenomenon in our model by estimating proportions of individuals in communities that have essentially undergone a natural immunization process, possibly due to exposures to low viral doses; but appropriate data is then needed to account for these natural vaccination rates [36].

It has always been the case that best practices require that we use the most appropriate models available at the time for assessing management options. In this vein, our model provides a useful tool at this time for understanding how reductions in contact rates of community members with infected individuals may bring the current West African EVD outbreak under control. It also helps us understand how effective vaccination programs could be, should a suitable vaccine be released. Additionally, our model exposes the limitations of the type of data available to support model fitting at this time. Our analyses suggest that regional campaigns to educate individuals on risky behaviors, detect cases more rapidly, isolate infected individuals more diligently, and deploy a vaccination program as soon as logistically feasible are all very important in moving towards extinguishing the epidemic within different countries, provided movement of infected individuals among countries can be detected and cases effectively isolated.

## Supplementary Material

Here we provide more details on the methods of data collection, mathematical equations used in the model formulation, and computational algorithms used in the simulations. We also provide information on the baseline parameters used in the simulations, as well as additional simulations results to demonstrate the sensitivity of model predictions to changes in baseline parameter values, including the vaccination studies. Finally, we provide daily resolution incidence data of the weekly resolution incidence data reported in the main text.

## Figures and Tables

**Table 1:** Summary of results from 20 simulations of model using the baseline data (Table S1).

Run number	Cases	Length (days)	*R*_0_ (*N*)^*^ 1–50	*R*_0_ 51–100	*R*_0_ 101–150	*R*_0_ 151–200	*R*_0_ HCW	Total *R*_0_
17	3627	235	2.48 (26)	1.71	0.93	0.51	0.37	1
3	2949	220	2.54 (25)	1.68	0.91	0.54	0.37	1
6	2236	229	2.41 (18)	1.64	0.92	0.53	0.34	1
8	1975	201	2.21 (19)	1.63	0.93	0.49	0.36	1
16	1658	240	2.33 (18)	1.62	0.87	0.51	0.46	1
9	1598	212	2.00 (20)	1.63	0.89	0.51	0.42	1
12	1456	232	2.55 (13)	1.59	0.91	0.54	0.42	1
11	1018	222	3.17 (7)	1.57	0.89	0.61	0.38	1
**Mean ≥1000**	**2065**	**224**	**2.46**	**1.63**	**0.91**	**0.53**	**0.39**	**1.00**

4	790	222	2.11 (11)	1.55	0.87	0.51	0.32	1
15	742	214	2.33 (7)	1.6	0.97	0.52	0.375	1
10	682	200	2.33 (5)	1.74	0.86	0.45	0.37	1
18	501	213	1.75 (5)	1.61	0.87	0.52	0.43	1
19	454	198	1.76 (9)	1.47	0.91	0.40	0.39	1
1	273	203	1.75 (8)	1.39	0.91	0.50	0.40	1
5	235	224	1.50 (4)	1.71	0.88	0.60	0.46	1
0	128	177	1.67 (6)	1.31	0.82	0.44	0.20	0.99
**Mean <1000**	**476**	**206**	**1.90**	**1.55**	**0.89**	**0.49**	**0.37**	**1.00**

Index cases that fail to cause outbreaks								
2, 7, 13, 14	1-2	16–28	NA	NA	NA	NA	NA	NA

^*^ *N* is the number of individuals in the offspring distribution use to calculate *R*_0_. Over subsequent intervals that are 50 units of time apart, the numbers of individuals in the offspring distribution are much larger when the number of cases exceeds 1000 (a couple to several hundreds) and hence estimates for these simulations are less variable across runs.
