# Supplementary material for: Tactics and Strategies for Managing Ebola Outbreaks and the Salience of Immunization
Source: Comput Math Methods Med. 2015 Feb 10;2015:736507. doi: 10.1155/2015/736507 (PMC4338386; doi:10.1155/2015/736507)
Supplement: Supplementary file 1 — Here we provide more details on the methods of data collection, mathematical equations used in the model formulation, and computational algorithms used in the simulations. We also provide information on the baseline parameters used in the simulations, as well as additional simulations results to demonstrate the sensitivity of model predictions to changes in baseline parameter values, including the vaccination studies. Finally, we provide daily resolution incidence data of the weekly resolution incidence data reported in the main text. [file 736507.f1.pdf]

Supplementary Online Information
Getz et al. Tactics and Strategies for Managing Ebola Outbreaks …

A. Methods

S1. Data

S1.1. Suspected Patients of Ebola Viral Disease (EVD) infection.

Blood samples were obtained from symptomatic suspected cases of Ebola fever for EVD active infection confirmation (WHO criteria). Patients were hospitalized at the Ebola Case Management Center in Kenema (Dr. S.H. Khan, head of the unit) of Kenema Government Hospital (Kenema District) and, lately at the Kailahun Ebola Case Management Center held by Doctors Without Borders (MSF) (Kailahun District). Patient's demographic and clinical data were recorded on the laboratory request forms. This work, strictly made for medical diagnostics, was done as part of the public health emergency response of the Ministry of Health and Sanitation with regards to the ongoing outbreak in Sierra Leone; informed consent was not obtained.

S1.2. Laboratory diagnostic assay.

Real time RT-PCR (rtRT-PCR) for Ebola virus nucleotide detection and characterization: Blood samples were processed at the Kenema Government Hospital, Viral Hemorrhagic Fever laboratory including high containment facilities for sample inactivation (BSL3), RNA extraction and PCR chains. Briefly, viral RNA was extracted from 140 μl of serum using QIAmp viral RNA kit (Qiagen) followed by rtRT-PCR as presented in detail elsewhere (Ebola Zaire rRT-PCR TaqMan® Assay, Naval Medical Research Center, U.S. Department of Defense. 2014). Reagents, supplies and equipment were provided by the Critical Reagents Program, USAMRIID and NIH.

S1.3 Epidemiologic Investigations and Field data recording.

Data, including demographic status (nuclei family), place of living and travel history were gathered on suspected patients through interviews of both suspected cases and relatives. After laboratory positive diagnosis of EVD, confirmed patients were isolated (dedicated health structure) and contact tracing were conducted on affected families, inhabitants of villages and other recently visited friend and families. Also attendants of funerals, public health authorities, and hospital staff members were investigated whenever required and data recorded for each examined person.

S2. Transmission Chain Model

The model depicted in Figure 1 (main text) was developed using the NOVA software platform. This software package can be downloaded at http://www.novamodeler.com/ and the model can be run on MacOS, Windows and Linux systems by opening the file

EbolaTransmissionChain.nva, made available below, using the NOVA platform. The mathematical structure of the model it depicted in Figure 1 (main text), the assumptions and equations provided in S2.1, and the parameters used in the simulation discussed in S2.2, while the actual code can be seen by opening the file EbolaTransmissionChain.nva using the NOVA software platform.

S2.1 Ebola Simulation Model Structure:

i. Four types of individuals (great oversimplification) two of which can be in states “Exp” (exposed and infected but not year infectious) or “Inf” (infectious). Note: post Inf individuals are recorded as dead or recovered, where the latter are likely immune but no longer accounted for in the model since the proportion of such individuals is taken to be considerable less than 1% of the susceptible individuals
Type=U: an infected individual that is not being treated, Exp then Inf
Type=T: an infected individual that is being treated, Inf only
Type=H: an infected healthcare worker; Exp then Inf
Type=V: an immune (vaccinated) individual, with prevalence v in the population

ii. Two time scales
a.) Global variable t, applies to whole population: t=0 is first or index case, $t=T$ the first time after t=0 that human-to-human transmission is no longer possible
b.) Local variable s, applies to an individual U or H: s=0 is the point at which an individual is infects (e.g. $s=t-t_1$ implies the individual became infected at t_1).

iii. Course of infection (type U and H specific values)
Transmission at s=0; latency over $[0, s_1)$ (Exp state); infectious over $[s_1, s_2)$ (Inf state); death with probability d at time s_2 or on the road to recovery and no longer infectious.

iv. A global time-dependent Poisson parameter $\lambda(t)$ is used to estimate the number of individuals that each type U agent infects each day to produce a new type U agents, each with probability $(1-v(t))$, where v is the vaccination prevalence. Assumption: U’s and only U’s create new U’s. The form of this dependency in terms of for parameters $0<\lambda_{\min}<\lambda_{\max}$, $c_1>0$, and $c_2>0$:

$$\lambda(t) = \lambda_{\min} + \frac{\lambda_{\max} - \lambda_{\min}}{1 + (t / c_1)^{c_2}}$$

while the form for $v(t)$, for parameters $0<v_{\min}<v_{\max}$, $c_7>0$, and $c_8>0$: is (see Figure 1, main text)

$$v(t) = v_{\min} + \frac{(v_{\max} - v_{\min})(t / c_7)^{c_8}}{1 + (t / c_7)^{c_8}}$$

v. A type U can then be transformed into a type T at time s^* on $[s_1, s_2)$ with probability $0 \leq \tau(s,t) \leq 1$. T's can only arise from U's. For simplicity, in our simulations we set $s_2=8+8=16$. The function $\tau(s,t)$ for parameters $c_3>0$, $c_4>0$, and $c_5>0$ is:

$$\tau(s,t)=\begin{cases} 0 & s \leq s_1, t \geq 0 \\ \dfrac{\left((s-s_1)t/(c_3t+c_4)\right)^{c_5}}{1+\left(s-s_1)t/(c_3t+c_4)\right)^{c_5}} & s_1 \leq s \leq s_2, t \geq 0 \end{cases}$$

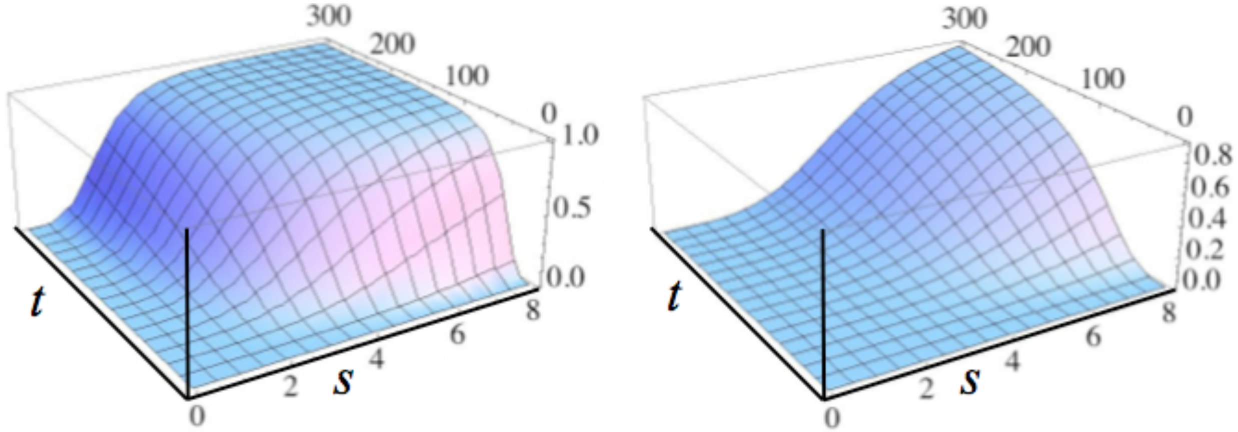

Figure S1. The function $\tau(s,t)$ plotted for values $c_5=4$, and: Left panel, $c_3=1$, $c_4=250$; Right panel, $c_3=3$, $c_4=500$.

vi. A constant Poisson parameter λ_{HCW} is used to estimate the number of infected H's created by T's for each remaining day on $[s^*= s_1, s_2)$. Note H's can only be created by T's. For simplicity, we have set $\lambda_{HCW} = \lambda_{min,}$ but in general this will not be true.

vii. The probability that an infected individual dies at time s_2 is given by $0 \leq d \leq 1$, if the individual is in state T at local time s_2 and is given by $de^{-c_6(s_2-s^*)}$ otherwise (which reduces to d when $s_2 = s^*$), for some $c_6>0$. For simplicity, we set $c_6=0$ and we focus in the incidence process (transmission) rather then the mortality process since mortality rates are currently uncertain as different treatment strategies are tried out using

viii. For purposes of transmission and death, healthcare workers (individuals in state H) are essentially T's from day $s^*= s_{1:}$ that is H's only create new H's each day on $[s^*= s_1, s_2]$ and die with probability $de^{c_6(s_2-s_1)}$. For simplicity we set $c_6=0$, particularly since our model focus on incidence rather than mortality rates.

ix. For infected individuals that do not die, some residual transmission probability is still possible after time s_2, but for simplicity we assumed this to be insignificant and hence set this probability to zero.

S2.2. Ebola Simulation Model Parameter Values:

The set of baseline parameters used to run the model are listed in Table S1, with alternative values used in several runs, as discussed in the main text, listed in parentheses.

Table S1. Baseline parameter values and alternatives (alts.)

**Function**	**Symbol**	**Designation**	**Value (alts.)**	**Comment**
**1. Poisson risk rate $\lambda(t)$ of new infection per infective per day**	λ_{min}	minimum value	0.05	range fitted
	λ_{max}	maximum value	0.30 (0.23)	range fitted
	c_1	community response (learning) parameter	100 (200, 350) days	response input
	c_2	switch rate control	4	
**2. Probability $v(t)$ that infected person has been vaccinated**	v_{min}	minimum value	0	response input
	v_{max}	maximum value	0 (0.05, 0.1, 0.2)	
	c_7	gear up rate	50 days	
	c_8	switch rate control	2	
**3. Transmission chain**	s_1	length of incubation period	8 days	Ref*, Table 2 est. median of 6.23 days
	s_2	length of infectious period	8 days	Ref*, Table 2 est. median of 8.66 days
**4. Probability $\tau(t,s)$ each day of U_{INF} being isolated**	c_3	scaling parameter 1	3 (1)	response input: See Fig. S1
	c_4	scaling parameter 2	500 (250)	
	c_5	switch rate control	4	
**5. Probability of death $d(s)$ as a function of days treated**	d_0	untreated death prob	0.5	current best rounded approx.
	c_6	damped exponential decrease of prob of death	0	response input

*Stadler, et al,, *Plos Current Outbreaks*, Oct 6, 2014 (see full ref in main text)

S3. Additional Results

Table S2. Summary of results from 20 simulations of model using the baseline data (Table S1) with the alternative values for $\lambda(t)$ (the lower R_0 rates in this case, compared with Table 1 in the main text, are expected because of the reduction in the value of λ_{max})

Run #	Cases	Length (days)	R_0 1-50	R_0 51-100	R_0 101-150	R_0 151-200	R_0 HCW	Total R_{0f}
7	7958	343	2.22	1.68	1.3	0.96	0.72	0.39
12	3601	313	2.08	1.76	1.33	0.95	0.67	0.43
19	3284	366	2.14	1.66	1.34	0.98	0.72	0.41
13	3001	307	2.11	1.8	1.23	0.95	0.67	0.35
18	1826	284	1.67	1.61	1.3	0.94	0.71	0.38
15	1646	287	1.89	1.3	1.35	0.94	0.66	0.35
9	1552	326	1.4	1.68	1.31	0.94	0.74	0.4
2	1356	286	1.33	1.78	1.32	1	0.71	0.35
Mean ≥1000	2643	310	1.86	1.68	1.30	0.95	0.68	0.37
1	964	273	1.75	1.62	1.29	0.93	0.57	0.43
14	583	314	1.67	1.55	1.23	0.99	0.57	0.47
10	479	285	1.43	1.35	1.39	0.92	0.75	0.48
6	224	252	1.50	1.25	1.10	1.11	0.60	0.46
16	53	205	2.00	1.05	0.85	0.78	NA	0.50
1	964	273	1.75	1.62	1.29	0.93	0.57	0.43
Mean <1000	461	266	1.67	1.36	1.17	0.95	0.62	0.47
Index cases that fail to cause epidemic								
3,4,5,8,11	1-4	17-56	NA	NA	NA	NA	NA	NA

Table S3. Data and Model Estimates of Ebola outbreak growth rates during Sept 2014.

	Dates/Day	Cases	Approximate doubling time
**Data***	Aug 25	3071	
	Sept 21	6242	26-27 days
**Model****	111	2995	
	130	6127	28-29 days

*From Table at url: http://en.wikipedia.org/wiki/Ebola_virus_epidemic_in_West_Africa

**From one baseline run with the community response (learning) parameter equal to 350

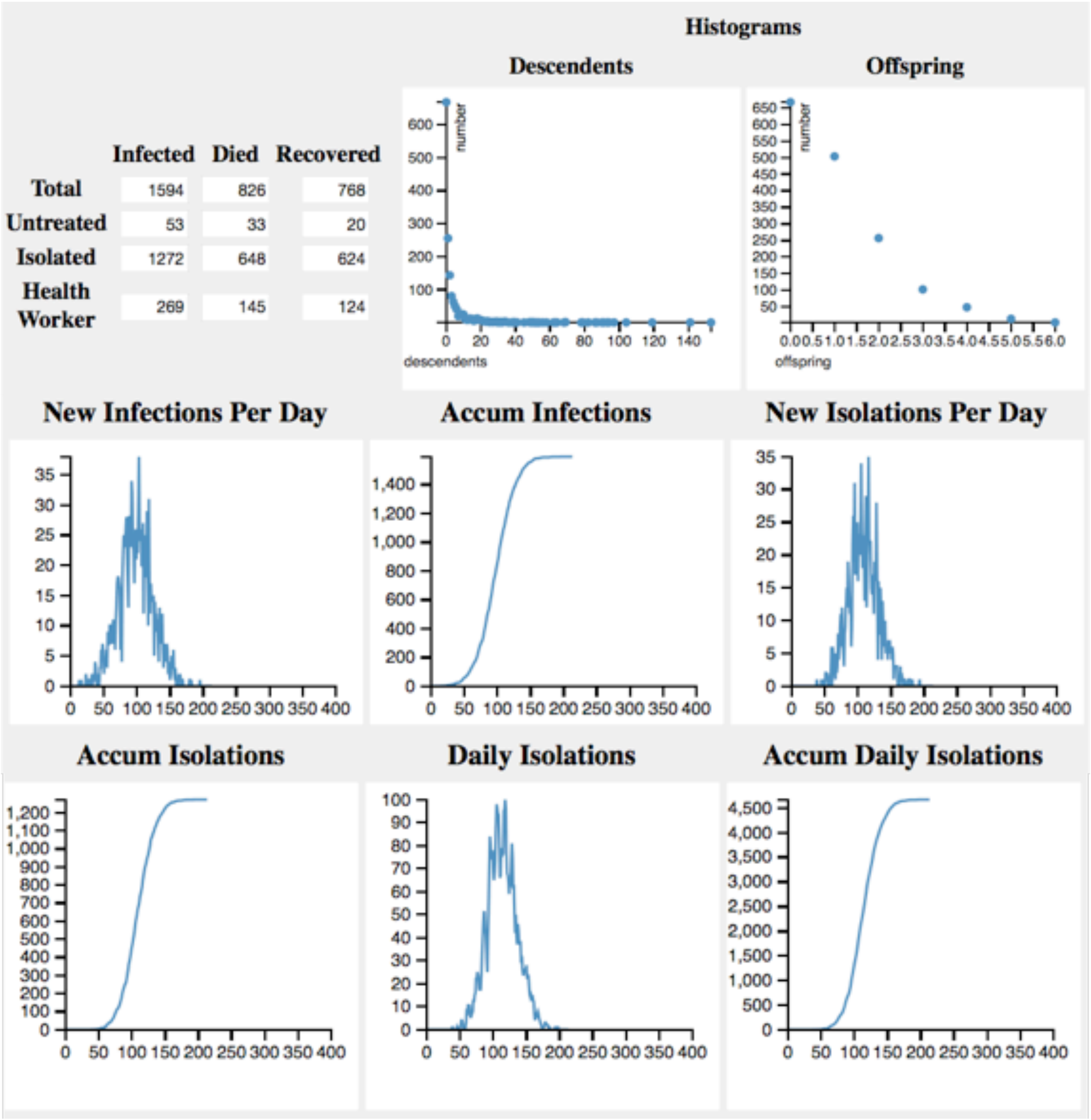

Figure S2. A collage of screen shoots from a webpage running our Ebola model web provides a panel of values (top left) on the size of the epidemic and graphs of outputs, as labeled. The histograms (top two panels) of the number of descendants that each case ultimately spawns (e.g. 650 cases do not infect anyone, while the tail of the descendants distribution has been truncated to 130, although the largest entry would be the total number of cases, i.e.1594, for the index case), as well as the next generation of offspring (the maximum number of cases that any case infects in the next generation is 6), have not been normalized to frequencies, but are actual numbers.

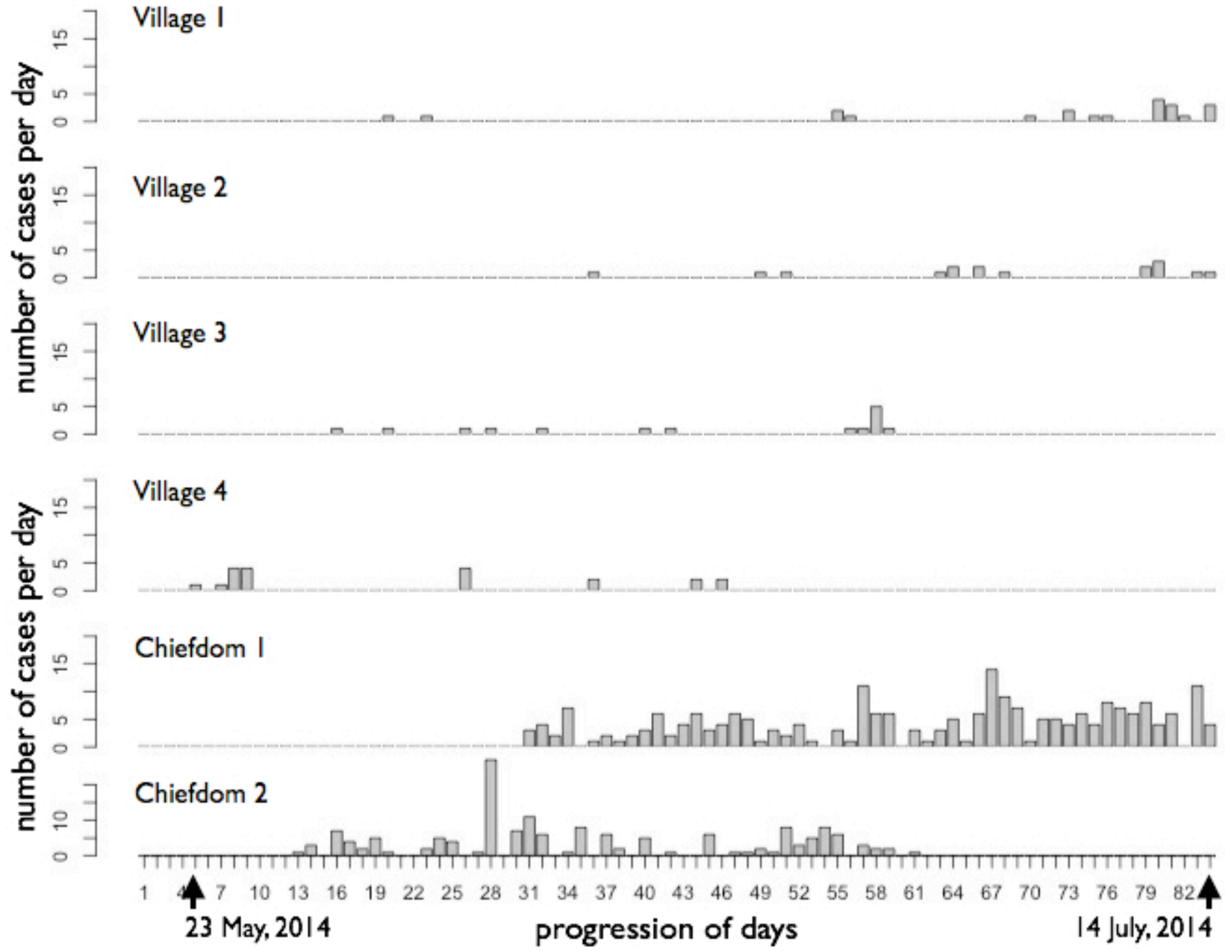

Figure S3. Number of cases per day recorded in 6 different local areas over the indicated time spans. The method for collecting these data is described in the Methods Section above.

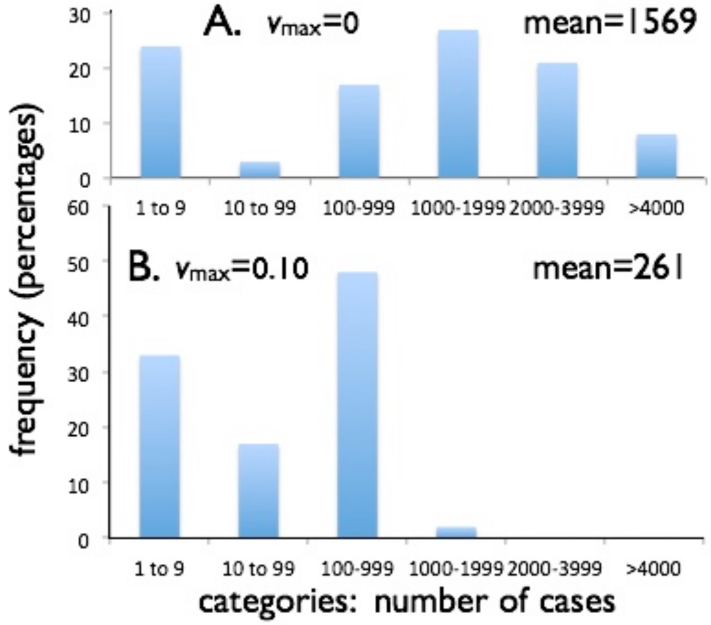

Figure S4. A-B: Histograms (percentages in each size class sum to 100) of epidemic sizes (number of cases) over 100 repeated simulations, using the alternative parameters (Table S1) with values for v_{max} as specified. **A**: When v_{max}=0 (no vaccination), outbreaks range from >4000 through a mode of 1000-1999, a mean of 1569 cases and a relatively high proportion (24%) of fadeouts (category 1-9 case bar). **B**: When v_{max}=0.10, very few outbreaks exceed 999 and the mean is 261 cases.
